# Cannabidiol Reduces Inflammatory Lung Damage After Meconium Aspiration in Newborn Piglets

**DOI:** 10.3389/fped.2022.862035

**Published:** 2022-06-06

**Authors:** Luis Arruza, Lorena Barata, Eva Vierge, Maria José Rodríguez, Aaron Del Pozo, William Hind, José Martínez-Orgado

**Affiliations:** ^1^Department of Neonatology, Hospital Clinico San Carlos—Instituto de Investigación Sanitaria San Carlos (IdISSC), Madrid, Spain; ^2^GW Research Ltd., Cambridge, United Kingdom

**Keywords:** meconium aspiration syndrome (MAS), cannabidiol, inflammation, piglet, translational research

## Abstract

**Aim:**

To assess the effects of cannabidiol (CBD) on lung damage in a piglet model of meconium aspiration syndrome (MAS).

**Materials and Methods:**

Meconium aspiration syndrome was modelled in newborn piglets *via* intratracheal instillation of 20% meconium in saline collected from healthy newborn humans. Piglets were treated i.v. with 5 mg/kg CBD (MAS + CBD) or Vehicle (MAS + VEH) 30 min after MAS induction and monitored for 6 h. Ventilated piglets without meconium instillation served as controls (CTL). Ventilatory and haemodynamic monitoring, histological and biochemical studies assessed the effects of treatment.

**Results:**

Post-insult administration of CBD reduced MAS-induced deterioration of gas exchange, improving respiratory acidosis (final pH 7.38 ± 0.02, 7.22 ± 0.03 and 7.33 ± 0.03 and final pCO_2_ 39.8 ± 1.3, 60.4 ± 3.8 and 45.7 ± 3.1 mmHg for CTL, MAS + VEH and MAS + CBD, respectively, *p* < 0.05). These beneficial effects were obtained despite the less aggressive ventilatory settings required for CBD-treated animals (final minute volume 230 ± 30, 348 ± 33 and 253 ± 24 mL/kg/min and final Oxygenation Index 1.64 ± 0.04, 12.57 ± 3.10 and 7.42 ± 2.07 mmHg for CTL, MAS + VEH and MAS + CBD, respectively, *p* < 0.05). CBD’s beneficial effects on gas exchange were associated with reduced histological lung damage, reduced leucocyte infiltration and oedema (histopathological score 1.6 ± 0.3, 8.6 ± 1.4 and 4.6 ± 0.7 points for CTL, MAS + VEH and MAS + CBD, respectively, *p* < 0.05), as well as reduced TNFα production (0.04 ± 0.01, 0.34 ± 0.06 and 0.12 ± 0.02 A.U. for CTL, MAS + VEH and MAS + CBD, respectively, *p* < 0.05). Moreover, CBD improved blood pressure stability (final mean blood pressure 74.5 ± 0.2, 62.2 ± 6.2, and 78.67 ± 4.1 mmHg for CTL, MAS + VEH and MAS + CBD, respectively, *p* < 0.05).

**Conclusion:**

Cannabidiol reduces histologic lung damage and inflammation in a piglet model of MAS. This translates into improved gas exchange and blood pressure stability.

## Introduction

Approximately 12% of births are accompanied by foetal passage of meconium *in utero* (1, 2). Of these, 4% are complicated by the development of meconium aspiration syndrome (MAS) in the newborn, a potentially devastating respiratory condition with high associated morbidity and mortality (3). The initiation of foetal inspiratory efforts or ventilation with positive pressure during resuscitation, together with the onset of spontaneous breathing in the newborn, causes the meconium present in the airway to produce partial or complete airway obstruction leading to the coexistence of areas of atelectasis and emphysema. Atelectasis causes ventilation-perfusion (V/Q) mismatch, whilst alveolar overdistention explains the high incidence of air-leak syndrome in this condition. *In vitro* studies on calf lung surfactant have shown that meconium also inactivates surfactant in a dose-dependent manner (4). Furthermore, meconium is a highly irritative substance that induces lung inflammation, which plays a key role in the pathophysiology of MAS-induced lung damage, increasing parenchymal oedema, impairing endothelial function, and spreading the damage to lung areas not exposed to meconium (2, 5, 6). Post-resuscitation reoxygenation contributes to tissue damage, inflammation and oxidative stress with damaging consequences (1, 2). One of them, of crucial importance for clinical management and survival, is pulmonary hypertension (PH), which causes severe hypoxemia and may negatively affect myocardial function and cardiac output (1). The combination of all these mechanisms translates clinically into hypoventilation, hypoxemia and tissue hypoperfusion, with the need for aggressive mechanical ventilation, high concentrations of oxygen, inhaled nitric oxide (iNO), haemodynamic support and extracorporeal membrane oxygenation (ECMO) in the most severe cases (3).

There is no specific treatment of MAS other than lung lavage with surfactant, but the efficacy and safety of this procedure is controversial (1). Surfactant without lavage and iNO offer some beneficial effects in selected cases (7). Thus, management is mostly supportive, dealing with ventilatory and hemodynamic problems, and preventing complications such as brain damage (7). Steroids did show some promise in preclinical studies as a therapeutic tool against MAS-induced inflammation, but this failed to translate into clear benefits in randomised controlled trials (RCT) (5). In addition, steroids increase the risk of infectious diseases, which can be a concerning complication (5). This, together with the concern for the deleterious effects on neurodevelopment associated with the use of steroids in the neonatal period, make such therapy problematic (7).

Cannabidiol (CBD) is a non-euphoric phytocannabinoid, and its administration across a range of animal models of disease is known to be associated with anti-inflammatory properties (8) including decreased lung inflammation in a murine model of LPS-induced lung damage (9) and reduced oxidative stress (10). Further, studies from our group have demonstrated that CBD 1 mg/kg i.v. was associated with reduced HI brain-induced inflammation-mediated lung damage in piglets (11). The mechanisms of action of CBD are not fully elucidated, but preclinical studies suggest that CBD possesses a diverse polypharmacology including antagonism of G protein-coupled receptor 55, desensitization of transient receptor potential cation channel subfamily V member 1 and activation of 5-HT_1*A*_ receptors (12, 13). Despite the lipophilic nature of CBD, our group has demonstrated that CBD can be administered by i.v. route using an emulsion of CBD on saline, ethanol and Solutol*^R^* (11, 14–16). Administration of CBD 1 mg/kg by i.v. bolus to newborn piglets leads to serum C_*max*_ = 263 ± 41 ng/mL with T_*max*_ = 0.15 h and t1/2 = 1.09 ± 0.27 h (14, 16).

This study aims to test the effects of CBD in a model of another inflammation-based lung disease, MAS.

## Materials and Methods

The experimental protocol met European and Spanish regulations for protection of experimental animals (86/609/EEC and RD 53/2013) and was approved by the Ethics Committee for Animal Welfare from the University Hospital Puerta de Hierro Majadahonda (Madrid, Spain) and Hospital Clínico San Carlos (Madrid, Spain).

### Experimental Protocol

#### Surgical Preparation

Two- to four-day-old male Landrace-White large piglets were intubated under 5% sevoflurane anaesthesia and then mechanically ventilated (Babylog8000, Dräger, Germany) under sedoanalgaesia and paralysis by continuous infusion of fentanyl citrate (Fentanest*^R^*, Kern Pharma, Spain) at 3 μg/kg/h and vecuronium bromide (*^R^*, Schering-Plough, NJ) at 0.6 mg/kg/h. In each animal an indwelling catheter was placed in the right jugular vein to infuse dextrose and saline 50% at a rate of glucose of 4 mg/kg/min. Cardiac output (CO), heart rate (HR) and mean arterial blood pressure (MABP) were monitored (PiCCO Plus, Pulsion) by a femoral artery indwelling catheter (Ominare CMS24, HP, Göblingen, Germany). Ventilatory parameters (by the ventilator’s computerised pneumotachography) and O_2_ saturation (SO_2_) (Ohmeda 5250 RGM, Louisville, CO) were continuously monitored. Body temperature, monitored using a rectal probe introduced 6 cm into the rectum, was maintained at 37.5–38.5°C using a water mattress (TecoTherm, Inspiration-Healthcare, West Sussex, United Kingdom). Arterial blood gases and glycaemia were assessed hourly throughout the experiment and maintained within normal limits. Finally, cerebral and systemic regional oxygen saturation (crSO_2_ and srSO_2_, respectively) were continuously recorded using a near-infrared spectroscopy (NIRS) device (INVOS oximeter, Covidien, Mansfield, MA, United States) with neonatal sensors (OxyAlert NIR Sensor, Mansfield, MA, United States) placed on the forehead and on the upper right side of the abdomen over the liver.

#### Meconium Aspiration Syndrome Induction

The model was based on that described by Davey et al. (17). Meconium was collected the day of birth from healthy newborns at the maternity ward of Hospital Clínico San Carlos, Madrid, Spain, and then diluted to a 20% mixture in sterile saline at room temperature, manually stirred and filtered through a gauze to remove large particulate matter.

After a 30 min period of stabilization, 3 mL/kg of the mixture, divided into two aliquots, was instilled through the intratracheal tube to induce MAS. The goal was to have SO_2_ fall below 92%. If the goal was not achieved after the second aliquot, an additional 1 mL/kg dose of meconium 20% was instilled, which was necessary only once in a piglet from the vehicle-treated group.

#### Drugs

Plant-derived highly purified CBD was supplied by GW Research Ltd. (Cambridge, United Kingdom) as a preformulated 3 mg/mL solution, and a corresponding vehicle solution was also supplied by GW.

#### Treatment

Thirty min after meconium instillation the piglets were randomly assigned to receive i.v. vehicle (MAS-VEH, *n* = 14) or CBD 5 mg/kg (MAS-CBD, *n* = 6), further diluted in saline. MAS-VEH group was more heavily weighted because of the high expected mortality. The total volume of administration for CBD or vehicle was 10 mL, which was infused by syringe pump over 15 min. Treatment was administered by a researcher who did not participate in the subsequent management of the piglet; thus, the researchers involved in piglet care were blinded to the treatment assignment.

#### Follow-Up

The piglets were ventilated and monitored for 6 h. Based on previous studies (11), starting ventilator setting were PEEP 5 cm H_2_O, ventilator rate 30 bpm and Vt 8 mL/kg. Ventilator settings were then changed as required to maintain SO_2_ over 92% and pCO_2_ between 35 and 55 mmHg. The approach to keep SO_2_ over 92% was: first, to increase FiO_2_ 10%; if no effect, to increase PEEP 10%; if no effect, to increase FiO_2_ again, etc. The approach to keep CO_2_ 35–45 was as follows: to reduce CO_2_: first, to increase PIP by 1 cm H_2_O steps in case of a drop in Vt; if no effect, then to increase ventilator rate 5 bpm; if no effect, then to increase PEEP in 1 cm H_2_O steps, etc. To increase CO_2_, same steps backwards. MABP was over 30 mmHg throughout the experimental period in all cases with no need for inotropic drugs.

Control animals were similarly managed piglets but without meconium instillation (Ventilated controls, CTL, *n* = 6). CTL piglets did not receive intratracheal saline because saline instillation is detrimental for airway and lung parenchyma.

#### Sampling

Six hours after meconium instillation, or the equivalent period in CTL, piglets were humanely killed by i.v. bolus of 20 mEq potassium chloride (Fresenius Kabi, Spain) and their lungs were manually inflated using a pediatric bag and rinsed with saline. Samples from upper, middle-anterior and lower lobes from left lung were snap frozen in isopentane and stored at –80°C for biochemical studies. Then the right lung was fixed with 4% paraformaldehyde for histological studies, which were conducted in the middle-anterior lobe, an area not affected by atelectasis related to the supine position of the piglets.

### Lung Damage Assessment

#### Histological Studies

Paraffin-embedded sections from the anterior segment of the right lung were stained with haematoxylin-eosin. Two experienced researchers blinded to the experimental groups performed a four-point, semi-quantitative, severity-based scoring system (negative = 0, slight = 1, moderate = 2, severe = 3) assessing atelectasis, oedema, inflammation and other variables, as described in more detail elsewhere (11).

#### Western Blot Studies

Samples of frozen lung (50 mg) were used to determine TNFα concentration by Western blot. The procedure was based on that previously described in more detail elsewhere (14). Briefly, TNFα assays were performed with lung samples containing 20 μg of total protein. The proteins were transferred to polyvinylidene fluoride membranes (GE Healthcare; Buckinghamshire, United Kingdom). The membranes were blocked by overnight incubation in PBS-Tween (PBST) containing 5% non-fat dried milk at 4°C. The resultant blots were incubated with anti-TNFα antibody overnight at 4°C [mouse anti-pig TNFα 1:500 (14); R&D System, MA, United States] or rabbit polyclonal anti-β-actin (1:10,000, Sigma Aldrich, Spain) for 1 h at room temperature in PBST containing 5% non-fat dried milk. Finally, blots were incubated with anti-rabbit or anti-mouse HRP-labelled secondary antibodies (1:5,000, Santa Cruz Biotechnology, Dallas, TX, United States) for 1 h at room temperature. The peroxidase reaction was developed with an electrochemiluminescence (ECL) kit (GE Healthcare; Buckinghamshire, United Kingdom). Finally, the films were scanned and analysed using ImageJ 1.43u software (U.S. National Institutes of Health). Protein levels were normalised by β-actin loading and expressed as TNF-α/β-actin ratios.

### Statistical Analyses

Statistical analyses were conducted using GraphPad Prism 9.2.0. software (GraphPad Software LLC, CA). No outliers were found using the ROUT outlier test. Normality was assessed using the Shapiro-Wilk test and all data were found to be normally distributed; therefore, one- or two-way ANOVAs were used. One-way ANOVA was applied to compare results from histological and biochemical studies among groups. Two-way ANOVA with repeated measures was applied to test the effect of time (within subjects) and group (between subjects). In both cases, if main effects were statistically significant, *post-hoc* comparisons were performed by Holm-Šidák’s *post-hoc* test for multiple comparisons. Contingency (2 × 2) tables were studied using the *X*^2^ test. Results are expressed as mean (SEM). A *p* < 0.05 was considered significant.

## Results

### General Outcome

There were no differences among groups in age (2.5 ± 0.2, 2.7 ± 0.2 and 2.8 ± 0.2 days for CTL, MAS + VEH and MAS + CBD, respectively, *p* = 0.27) or weight (2.3 ± 0.1, 2.3 ± 0.1 and 2.7 ± 0.2 kg for CTL, MAS + VEH and MAS + CBD, respectively, *p* = 0.28) at the day of the experiment. Four out of 14 MAS + VEH piglets died (one from acute pneumothorax and three from acute myocardial infarction), which was consistent with mortality reported for the model (17). None of the 6 MAS + CBD piglets died, but one developed severe pneumothorax which required a thoracentesis. This difference was not statistically significant (*X*^2^ = 1.42, *p* = 0.21)

### Haemodynamics and Ventilation

**FIGURE 1:**
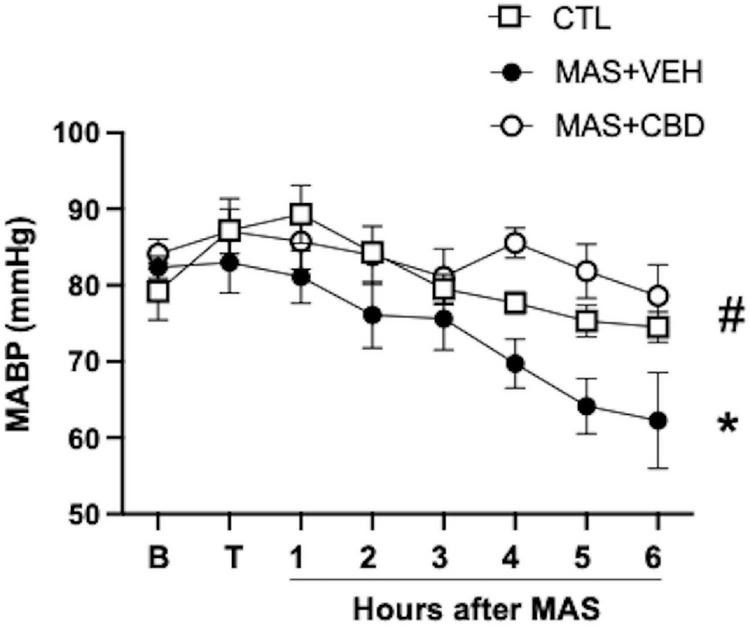
Change over time on mean arterial blood pressure (MABP) in ventilated control (CTL, *n* = 6) animals and piglets submitted to meconium aspiration (MAS) and then treated with i.v. vehicle (VEH, *n* = 10) or CBD (*n* = 6). B, basal (pre-MAS); T, treatment administered (30 min after MAS). Data presented as mean ± SEM. Two-way ANOVA with Holm-Šidák’s *post-hoc* test for multiple comparisons. (*) *p* < 0.05 vs. CTH, (#) *p* < 005 vs. MAS + VEH.

Data concerning haemodynamic and ventilatory parameters are described in Table 1. CO remained stable over the experimental period in all piglets. HR was elevated in MAS + VEH and MAS + CBD piglets, starting shortly after meconium instillation. Then, HR remained stable throughout the experimental period in MAS + CBD whereas in MAS + VEH it was a numerical trend to a decrease in HR which did not reach statistical significance. As a result, two-way ANOVA analysis revealed a significant effect for group but not for time. CBD treatment prevented the MAS-induced progressive fall in MABP (Figure 1). Results of the two-way repeated measures ANOVA revealed a significant effect of both time and group, with a trend to a progressive fall in MABP values over time, more dramatic in MAS + VEH than in the other groups. *Post-hoc* comparisons showed significant differences between MAS + VEH and CTL or MAS + CBD.

**TABLE 1:** Haemodynamic and ventilator parameters.

Parameter measured		CTL (*n* = 6)	MAS + VEH (*n* = 10)	MAS + CBD (*n* = 6)	2-way ANOVA
**Haemodynamics**					
CO (mL/Kg/min)	B	335.6 (29.1)	344.8 (18.5)	325.3 (21.9)	Time: *p* = 0.37
	T	360.1 (22.3)	368.3 (16.0)	320.3 (26.5)	Group: *p* = 0.05
	1	356.8 (26.7)	364,0 (22.0)	345.2 (34.0)	Interact: *p* = 0.9
	3	297.5 (25.2)	378.4 (23.8)	321.6 (32,5)	
	6	309.6 (26.7)	298.3 (31.9)	306.5 (43.5)	
			*	* #	
Heart rate (bpm)	B	160 (12)	193 (15)	201 (16)	Time:, *p* = 0.24
	T	177 (16)	215 (11)	225 (10)	Group: *p* = 0.00003
	1	177 (16)	221 (8)	249 (12)	Interact: *p* = 0.47
	3	175 (11)	209 (9)	249(10)	
	6	183 (13)	180 (11)	239(13)	
**Ventilation**					
			*	*	
FiO_2_	B	0.22 (0.01)	0.22 (0.008)	0.24 (0.02)	Time:, *p* = 0.0002
	T	0.22 (0.05)	0.56 (0.09)	0.51 (0.05)	Group: *p* = 0.01
	1	0.23 (0.04)	0.49 (0.07)	0.44 (0.07)	Interact: *p* = 0.99
	3	0.24 (0.06)	0.56 (0.09)	0.46 (0.07)	
	6	0.27 (0.08)	0.66 (0.11)	0.56 (0.12)	
			*		
SO_2_ (%)	B	96.7 (0.8)	97.2 (0.6)	97.2 (1.0)	Time: *p* = 0.01
	T	96.2 (0.9)	93.4 (0.7)	93.8 (0.9)	Group: *p* = 0.01
	1	96.5 (0.8)	94.2 (1.0)	97.8 (0.5)	Interact: *p* = 0.78
	3	98.5 (0.9)	92.2 (1.3)	94.5 (1.7)	
	6	94.2 (0.8)	81.3 (5.1)	90.1 (4.4)	
			*	*	
MAP (cm H_2_O)	B	7.2 (0.6)	7.8 (0.3)	8.4 (0.4)	Time: *p* = 0.00002
	T	7.3 (0.7)	10.8 (0.05)	9.5 (0.4)	Group: *p* = 0.01
	1	7.4 (0.4)	11.1 (0.5)	9.1 (0.4)	Interact, *p* = 0.92
	3	7.3 (0.6)	11.2 (0.5)	10.0 (0.8)	
	6	7.9 (0.8)	13 (1.0)	11.9 (2.3)	
Ventilator rate (bpm)	B	31.5 (1.7)	31.3 (1.3)	30.6 (1.6)	Time: *p* = 0.01
	T	32.3 (2.5)	31.3 (1.6)	30.8 (1.2)	Group: *p* = 0.88
	1	32.8 (3.1)	33.5 (1.8)	30.8 (1.2)	Interact: *p* = 0.59
	3	32.3 (2.5)	32.8 (1.7)	33.3 (3.1)	
	6	32.3 (2.5)	35.2 (2.3)	35.3 (3.3)	
PEEP (cmH_2_O)	B	5.0 (0)	5.0 (0)	5.0 (0)	Time: *p* = 0.0001
	T	5.0 (0)	5.1 (0.1)	5.1 (0.1)	Group: *p* = 0.27
	1	5.0 (0)	5.1 (0.1)	5.1 (0.1)	Interact: *p* = 0.16
	3	5.0 (0)	5.4 (0.3)	5.3 (0.3)	
	6	5.0 (0)	5.8 (0.3)*	5.7 (0.4)*	
			*	*	
OI (ratio)	B	1.5 (1.0)	1.6 (0.8)	1.7 (0.7)	Time: *p* = 0.0002
	T	1.5 (0.8)	7.1 (1.4)	6.5 (1.2)	Group: *p* = 0.003
	1	1.5 (0.9)	6.4 (1.2)	4.8 (1.3)	Interact: *p* = 0.99
	3	1.4 (1.9)	8.9 (2.1)	5.5 (1.3)	
	6	1.9 (0.9)	12.6 (3.4)	10.9 (3.9)	

Mean (SEM). B, basal; T, moment at which the treatment (VEH, vehicle; CBD, cannabidiol 5 mg/kg) was administered. 1, 3, 6: hours after meconium aspiration (MA). CO, cardiac output; SO_2_, arterial oxygen saturation; FiO_2_, oxygen fraction in the inspired air; MAP, mean airway pressure; OI, oxygenation index. (*) P < 0.05 vs. CTL, (#) p < 0.05 vs. MAS + VEH, all by two-way ANOVA with Holm-Šidák’s post-hoc test for multiple comparisons.

Meconium aspiration syndrome-induced lung damage was reflected by the more aggressive ventilator parameters required to maintain oxygenation, as illustrated by oxygenation index (OI = MAP × FiO_2_ × 100/PaO_2_), MAP and FiO_2_ requirements in MAS + VEH and CTL groups. These parameters were similar in MAS + VEH and MAS + CBD piglets (Table 1). Ventilator rate was similar in all groups, whereas PEEP values increased over time in both MAS groups to reach statistical difference vs. CTL 6 h after meconium instillation (Table 1).

**FIGURE 2:**
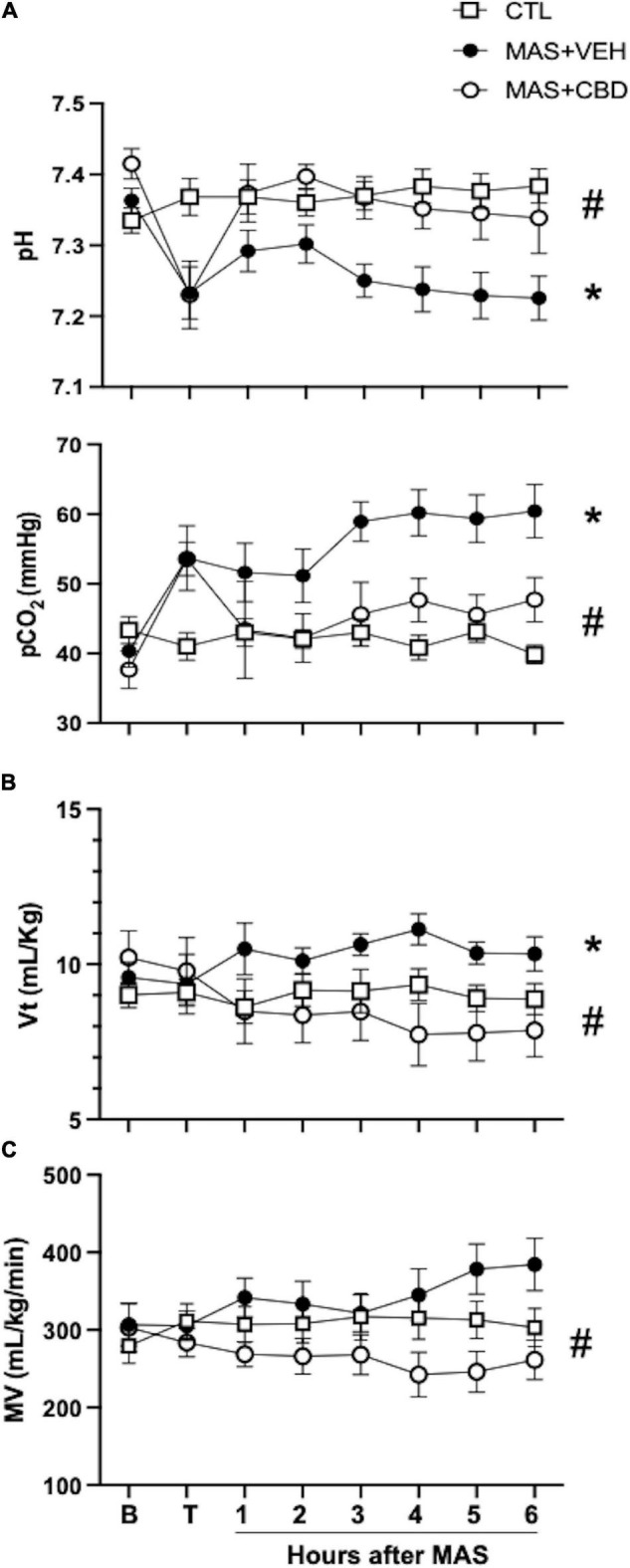
Changes over time on blood gases **(A)** and ventilator settings **(B,C)** in ventilated control (CTL, *n* = 6) animals and piglets submitted to meconium aspiration (MAS) and then treated with i.v. vehicle (VEH, *n* = 10) or CBD (*n* = 6). B, basal (pre-MAS); T, treatment administered (30 min after MAS); Vt, tidal volume; MV, minute volume. Data presented as mean ± SEM. Two-way ANOVA with Holm-Šidák’s *post-hoc* test for multiple comparisons. (*) *p* < 0.05 vs. CTH, (#) *p* < 005 vs. MAS + VEH.

Regarding the factors affected by ventilation, MAS led to progressive respiratory acidosis, as shown by the declining pH together with increasing pCO_2_ observed in MAS + VEH animals (Figure 2A). This effect was reduced by CBD treatment after MAS, leading to less elevated pCO_2_ levels and normal pH (Figure 2A). Results of a two-way repeated measures ANOVA revealed a significant effect of both time and group for pH and pCO_2_, supporting that respiratory acidosis evolved over time in MAS animals but differently in MAS + VEH and MAS + CBD animals.

It is noteworthy that the effects of CBD on pCO_2_ were achieved despite lower tidal volume (Vt values) and minute ventilation (MV values) in MAS + CBD piglets compared to MAS + VEH animals (Figures 2B,C). In this case, the two-way ANOVA only revealed differences for group, because those parameters changed from 3 h after MAS.

### Histological Study

**FIGURE 3:**
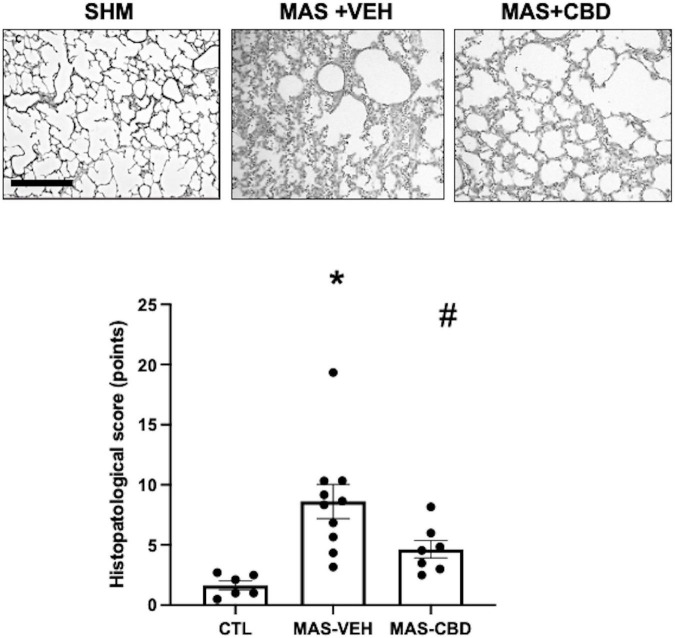
Histopathological score as assessed after haematoxylin-eosin staining in lung tissue from ventilated control (CTL, *n* = 6) animals and piglets submitted to meconium aspiration (MAS) and then treated with i.v. vehicle (VEH, *n* = 10) or CBD (*n* = 6). Top***:*** Representative light microphotographs. In lung from MAS-VEH animals there is interstitial leukocyte infiltration, haemorrhage and interstitial oedema, which was reduced by CBD treatment. Original magnification x200, bar: 200 μm. Bottom*:* quantification of lung damage by a severity score. Results are expressed as mean ± SEM of 6–10 animals. (*) *p* < 0.05 vs. CTL, (#) *p* < 0.05 vs. MAS + VEH by one-way ANOVA with Holm-Šidák’s *post-hoc* test for multiple comparisons.

Meconium aspiration syndrome + VEH piglets displayed profound lung damage with increased leucocyte infiltration, parenchymal oedema and haemorrhage (Figure 3). CBD treatment significantly reduced such histological features of lung damage (Figure 3).

### Cytokine Determination

**FIGURE 4:**
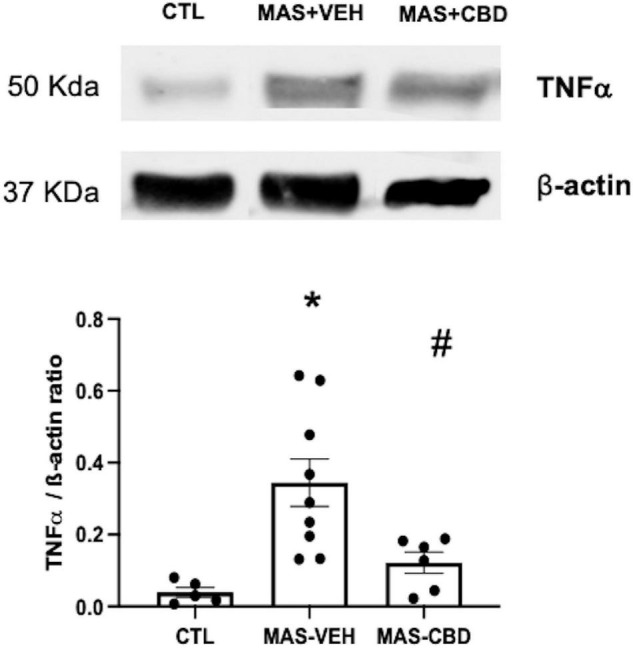
Concentration of TNFα as measured by western blot in lung tissue from ventilated control (CTL, *n* = 6) animals and piglets submitted to meconium aspiration (MAS) and then treated with i.v. vehicle (VEH, *n* = 10) or CBD (*n* = 6). Bars represent the mean (SEM) of 6–10 animals. Protein levels were normalised to β-actin loading and expressed as TNF-α/β-actin ratio. (*) *p* < 0.05 vs. CTL, (#) *p* < 0.05 vs. MAS + VEH by one-way ANOVA with Holm-Šidák’s *post-hoc* test for multiple comparisons.

Meconium aspiration syndrome increased TNFα levels in lung tissue, which was significantly reduced in MAS + CBD piglets (Figure 4).

## Discussion

In this study we demonstrate that post-insult administration of CBD exerts beneficial effects in a piglet model of MAS. A striking result was that CBD treatment after MAS exerted a beneficial effect on ventilation, with less elevated pCO2 levels and normal pH, but CBD did not improve oxygenation, with similar FiO2 requirements and OI in MAS + VEH and MAS + CBD piglets throughout the experimental period. Oxygenation problems in MAS are a consequence of impaired diffusion due to V/Q mismatch caused by different factors, including the presence of non-ventilated atelectatic areas as a result of mechanical obstruction of the airways by meconium and alveolar occupation by edema, inflammation or meconium, as well as thickening of the blood gas barrier caused by inflammation (1, 2). In MAS, V/Q mismatch is not homogeneous in lung parenchyma since meconium is not evenly distributed, leading to areas with more inflammation than others (1, 2). V/Q inequality disrupts the transfer of all gases in lung including O_2_ and CO_2_ (18). We speculate that the anti-inflammatory effect of CBD decreased the V/Q inequality derived from an uneven distribution of inflammation in the lung after MAS and thinning of the blood-gas barrier, resulting in improved gas diffusion. Such hypotheses are supported by the fact that CBD’s effects on pCO_2_ were achieved despite lower MV and Vt values in MAS + CBD piglets as compared to MAS + VEH animals. The different dissociation curves of O_2_ and CO_2_, however, determines that improvements of V/Q inequality results in improvements in ventilation earlier than in oxygenation (18). Increasing pulmonary blood flow in the presence of ventilation-perfusion inequality also improved ventilation before improving oxygenation (18). CBD improves pulmonary blood flow at doses that do not affect systemic hemodynamics (19) but this was not assessed in our experiments. Therefore, it is possible that a longer follow-up would be required to detect an improvement in oxygenation in our model. Hypoxemia after MAS can also be a consequence of secondary pulmonary hypertension (1, 2), but this condition was not assessed in our study.

Meconium aspiration syndrome-induced lung damage was demonstrated in the histological analyses, with increased leucocyte infiltration, parenchymal oedema and haemorrhage in MAS-VEH lungs, features that have been attributed to a meconium-induced inflammatory reaction in lungs (17). In agreement, we observed that histological injury was associated with increased production of TNFα, a well-documented finding in piglet models of MAS (5). CBD treatment reduced MAS-induced lung damage, reducing leucocyte infiltration, parenchymal oedema and haemorrhage. These beneficial effects were consistent with the prevention of MAS-induced increases in TNFα concentration by CBD. These results agree with previous studies demonstrating anti-inflammatory properties of CBD on the lung, in murine models of LPS-induced lung damage as well as in cerebral hypoxia-ischemia-mediated distant lung injury in newborn piglets (9, 11). The present results, however, are a more robust demonstration of the anti-inflammatory effect associated with CBD in the lung considering MAS is a lung disease with a particularly strong inflammatory component (2, 5, 6). Thus, although CBD shows some effects that could theoretically account for lung protection after MAS, such as the improvement of pulmonary blood flow at doses that do not affect systemic hemodynamics (19) and the modulation of oxidative stress (10), the paramount importance of inflammation in MAS pathophysiology points to the anti-inflammatory effects as the main component responsible for the protective effect of CBD in MAS. In fact, known anti-inflammatory substances such as steroids, in particular prednisolone and dexamethasone, administered shortly after meconium infusion in newborn pigs and rabbits, lead to similar beneficial effects, improving gas exchange, decreasing ventilatory pressures and reducing lung oedema (5). Therefore, CBD achieved similar anti-inflammatory-based beneficial effects to steroids without significant side effects.

In fact, CBD treatment prevents the MAS-induced progressive fall in MABP, which was significant in vehicle-treated piglets, although no supportive inotropic treatment was initiated since MABP did not fall below 30 mmHg. If the duration of this study was extended inotropic support may well have been required. This effect was similar to that reported for piglets after a hypoxic-ischaemic insult (14, 15). Such beneficial effects of CBD on blood pressure in two different neonatal models are reassuring and open an interesting possibility for CBD as a part of the treatment of hemodynamic instability in critically ill neonates. Interestingly, MAS + CBD piglets showed increased HR starting shortly after CBD administration, which remained stable throughout the experimental period. Such an effect was not observed in HI piglets receiving CBD 1 mg/kg i.v. (14, 15). It remains to be elucidated if this effect is a consequence of the higher dose of CBD used in the present study or if this difference resulted from using CBD in a different condition. The clinical relevance of this effect is likely to be limited, since it did not translate into hemodynamic instability with CO and MABP remaining unaffected.

Our study presents several shortcomings. The main limitation of our study is that MAS was induced at a postnatal age of 2–4 days, so the animals had probably completed cardiopulmonary transition by that time. In a more translational model MAS results from prenatal meconium aspiration during gasping after inducing asphyxia, which is likely to leads to lung injury and lung vessel reactivity changes more similar to the actual condition in human newborns (20). However, in our model piglets showed greater ventilatory and hemodynamic instability than lambs over a similar follow-up period (20), which might represent an advantage when the aim is to test the efficacy of a novel therapy. In this regard, although the presence of pulmonary hypertension was not assessed and consequently the effect of CBD on it could not be evaluated, its contribution to hypoxemia in our model was less likely. On the other hand, the role of surfactant inactivation in our model was not studied and thus the effect of CBD on this pathophysiological mechanism could not be assessed either. Finally, studies with longer time of follow-up would be necessary to get more insights into the mechanisms of CBD beneficial effects as well as the true translational value of the treatment.

The dose of CBD used in this study was based on the fact that 1 mg/kg CBD has demonstrated efficacy in mild- to moderate- models of HI in newborn rats, mice and piglets, but not in severe models of HI in newborn piglets (21). The model of MAS employed in this study produces a profound inflammatory insult so it was hypothesized that a higher dose of CBD might be required; furthermore, 5 mg/kg CBD has shown beneficial effects in rodent models of newborn and adult ischemic stroke (10). However, future studies could explore the potential efficacy of lower and higher doses of CBD in this model of MAS.

In conclusion, CBD administered after MAS induction in newborn piglets improved ventilation and respiratory acidosis with lower ventilatory requirements. These effects were associated with the reduction of histological lung damage in a manner related to modulation of inflammation. CBD treated animals were hemodynamically more stable without significant side-effects. This constitutes an asset for CBD when compared to other anti-inflammatory therapies (e.g., steroids). More studies are needed to further characterize the effects of CBD on MAS.

## Data Availability Statement

The raw data supporting the conclusions of this article will be made available by the authors, without undue reservation.

## Ethics Statement

The animal study was reviewed and approved by Ethics Committee for Animal Welfare from the University Hospital Puerta de Hierro Majadahonda (Madrid, Spain) and Hospital Clínico San Carlos (Madrid, Spain).

## Author Contributions

LA and JM-O contributed to the conception and design of the study and wrote the first draft of the manuscript. LA, LB, EV, MR, and JM-O performed the experiments. LA, LB, AD, and JM-O performed the biochemical and histological studies. JM-O and WH performed the statistical analysis and organized the database. WH edited the final version of the manuscript. All authors contributed to manuscript revision and read and approved the submitted version.

## Conflict of Interest

JM-O had a Research Agreement with GW Research Ltd. (Cambridge, United Kingdom) when these experiments were performed. WH was an employee of GW Research Ltd. (Cambridge, United Kingdom) where he received salary and held shares. The remaining authors declare that the research was conducted in the absence of any commercial or financial relationships that could be construed as a potential conflict of interest.

## Publisher’s Note

All claims expressed in this article are solely those of the authors and do not necessarily represent those of their affiliated organizations, or those of the publisher, the editors and the reviewers. Any product that may be evaluated in this article, or claim that may be made by its manufacturer, is not guaranteed or endorsed by the publisher.
